# Immunological Aspects of Aging

**DOI:** 10.1093/geroni/igaf122.817

**Published:** 2025-12-31

**Authors:** Anthony Covarubbias

## Abstract

The immune system undergoes significant changes and dysregulation during aging, leading to immune cell exhaustion and senescence, impaired tissue homeostasis, age-related chronic inflammation (inflammaging), and decreased pathogen immunity. This session will focus on the immunological aspects of aging and highlight new insights into the molecular and cellular mechanisms that drive immune dysfunction and inflammaging. Dr. Anthony J. Covarrubias (UCLA) will chair this session and also discuss recent discoveries that macrophages can undergo cellular senescence, adopting pro-inflammatory phenotypes that promote chronic inflammation and fibrosis in aging and metabolic disease. Dr. Emily Goldberg (UCSF) will explore the cross-talk between metabolism and immune cells during aging, and how this impacts tissue inflammation during aging. Dr. Duygu Ucar (The Jackson Laboratory) will present systems-level and single-cell genomic approaches that reveal molecular signatures of immune aging, including how biological sex, infection history, and epigenetic remodeling shape immune responses and vaccine efficacy across aging. Dr. Seokjo Kang (Cedars-Sinai Medical Center) will conclude by examining molecular mechanisms controlling microglial aging, including sex-specific differences in microglial gene expression, metabolism, and function, and how these changes contribute to neuroinflammation and cognitive decline. Together, these talks will explore cutting-edge approaches and recent discoveries in molecular and cellular biology, metabolism, and computational fields to enhance our understanding of immune aging and its systemic effects on the aging process. The session will also showcase emerging therapeutic strategies aimed at rejuvenating the immune system and extending health span.

